# Antibodies to Inhibitory Synaptic Proteins in Neurological Syndromes Associated with Glutamic Acid Decarboxylase Autoimmunity

**DOI:** 10.1371/journal.pone.0121364

**Published:** 2015-03-16

**Authors:** Nuria Gresa-Arribas, Helena Ariño, Eugenia Martínez-Hernández, Mar Petit-Pedrol, Lidia Sabater, Albert Saiz, Josep Dalmau, Francesc Graus

**Affiliations:** 1 Neuroimmunology Program, Institut d’Investigació Biomèdica August Pi i Sunyer (IDIBAPS), Barcelona, Spain; 2 Service of Neurology, Hospital Clinic, Universitat de Barcelona, Barcelona, Spain; 3 Institució Catalana de Recerca i Estudis Avançats (ICREA), Barcelona, Spain; 4 Department of Neurology, University of Pennsylvania, Philadelphia, Pennsylvania, United States of America; Charite - Universitätsmedizin Berlin, GERMANY

## Abstract

Antibodies to glutamic acid decarboxylase (GAD-ab) associate to different neurological syndromes. It is unknown if the diversity in syndrome association represents epitopes in different immunodominant domains or co-existence of antibodies to other proteins of the inhibitory synapsis. We examined the serum and CSF of 106 patients with anti-GAD related syndromes (39 cerebellar ataxia, 32 stiff-person syndrome [SPS], 18 epilepsy, and 17 limbic encephalitis [LE]). GAD65-ab titres were quantified by ELISA. Immunoblot was used to determine if the antibody-targeted epitopes of GAD65 and GAD67 were linear. A cell-based assay (CBA) with HEK293 cells expressing the GAD65 N-terminal, central catalytic domain, or C-terminal was used to investigate the immunodominant domains. Antibodies to GAD67, gamma-aminobutyric acid A receptor (GABAaR), glycine receptor (GlyR), GABAaR-associated protein (GABARAP), and gephyrin were determined with CBA. GAD-ab internalization was investigated using cultured rat hippocampal neurons. CSF GAD65-ab titres were higher in patients with cerebellar ataxia and LE compared to those with SPS (p = 0.02). GAD67-ab were identified in 81% of sera and 100% of CSF. GAD65-ab recognized linear epitopes in 98% of the patients and GAD67-ab in 42% (p<0.001). The GAD65 catalytic domain was recognized by 93% of sera, and the three domains by 22% of sera and 74% of CSF (p<0.001). Six patients had GABAaR-ab and another 6 had GlyR-ab without association to distinctive symptoms. None of the patients had gephyrin- or GABARAP-ab. GAD65-ab were not internalized by live neurons. Overall, these findings show that regardless of the neurological syndrome, the CSF immune response against GAD is more widespread than that of the serum and that there is no specific association between clinical phenotype and the presence of antibodies against other proteins of the inhibitory synapsis.

## Introduction

High levels of antibodies against glutamic acid decarboxylase (GAD-ab) have been reported in serum of patients with several neurological syndromes, including stiff person syndrome (SPS), cerebellar ataxia, epilepsy, and limbic encephalitis (LE), all of them characterized by neurological dysfunction of the GABAergic system [1–3]. The reason why some patients develop one neurological syndrome versus another is unclear. Neurological syndromes linked to GAD-ab were initially described in 1988 [4] but to date there are no large series or comprehensive studies comparing the spectrum and heterogeneity of the immune responses that occur in patients with diverse anti-GAD-associated syndromes. Studies addressing this issue are small or restricted to SPS, predominantly focused on the GAD65 isoform, or using only serum. In addition, it was postulated that in patients with GAD-ab and LE or seizures, these symptoms could be caused by more relevant autoantibodies against cell surface antigens and respond well to immunotherapy [5]. On the other hand, there are patients with LE and isolated GAD-ab that appear to have worse outcome [6]. Therefore, determination of whether patients with different anti-GAD associated syndromes have distinct underlying immune responses may have practical clinical implications.

The pathogenic significance of GAD65-ab is controversial. Some studies suggest they play a direct pathogenic role, but several lines of evidence suggest otherwise. First, GAD65-ab-positive neurological syndromes do not respond well to immunotherapy compared to those associated with antibodies against neuronal surface antigens [7,8], second, there is no correlation between antibody titres and disease severity [9], and third, there are no convincing animal models of the neurological disorders [10,11]. An important step towards proof of pathogenicity would be the demonstration that GAD-ab bind to live neurons, and after internalization reach the intracellular GAD isoforms.

To address all these questions, we examined serum or CSF of 106 patients with different anti-GAD associated neurological syndromes aiming to determine the repertoires of antibodies against the two GAD isoforms, the main immunodominant regions and linear or conformational structure of the epitopes, the presence of co-existing antibodies to other proteins or receptors of the inhibitory synapses, and whether GAD-ab were internalized by live neurons.

## Materials and Methods

### Patients and inclusion criteria

Patients were seen by the authors or referring physicians between December 1994 and April 2013. Serum or CSF were examined for autoantibodies in the laboratory of Neuroimmunology at the Institut d’Investigacions Biomèdiques August Pi i Sunyer (IDIBAPS), Hospital Clinic, Barcelona, Spain, or in the Department of Neurology, Hospital of the University of Pennsylvania, Philadelphia, USA. Inclusion criteria was the presentation of a neurological disorder associated with serum GAD65-ab detected by brain immunohistochemistry (this technique detects GAD-ab with radioimmunoassay (RIA) levels >2000U/mL; patients below these titres have diabetes (T1DM), but almost never neurological symptoms) [1] and confirmed by cell-based assay (CBA) of HEK293 cells expressing GAD65. Patients with a definite or possible paraneoplastic syndrome were excluded from the study [12].

Clinical information was obtained directly by the authors or provided by referring physicians, through questionnaires and telephone interviews. SPS included patients with classical forms, defined by stiffness and spasms predominantly involving the proximal aspect of the legs and lumbar muscles, and patients with partial forms, including stiff-limb syndrome or isolated lower or upper extremity stiffness [13]. Cerebellar ataxia was considered in patients who developed a cerebellar syndrome and fulfilled previously reported criteria (absence of alternative explanation and high GAD65-ab levels) [8]. LE was defined by the subacute onset of short-term memory loss, behaviour change, seizures, and involvement of the temporal lobes by imaging studies, or post-mortem examination [12,14]. Patients who developed isolated epilepsy without neuroimaging criteria of limbic involvement were classified as epileptic patients. When several GAD-associated neurological syndromes coexisted in a patient, the predominant syndrome by the time of diagnosis was used to classify such patient. Overall, 106 patients where included in the study. Four groups with different neurological syndromes were identified: 39 with cerebellar ataxia (CSF samples: 26), 32 with SPS (17), 17 with LE cases (10), and 18 with epilepsy (8). Table 1 summarizes the clinical characteristics of the study cohort.

**Table 1:** Basic clinical information of the cohort study.

	Cerebellar ataxia	Stiff-person syndrome	Isolated epilepsy	Limbic encephalitis
Patients, (CSF)	39 (26)	32 (17)	18 (8)	17 (10)
Female, (%)	32 (82)	29 (91)	15/17 (88)	12/15 (80)
Age (in years), median (range)	60 (32–79)	53 (5–77)	32 (9–67)	26 (12–49)
Overlapping syndrome	14 (9^a^+5^b^)	5 (3^b^+2^c^)	0	1^a^
T1DM at onset, (%)	12/32 (38)	14/29 (48)	4/9 (44)	2/6 (33)
Thyroiditis, (%)	18/30 (60)	7/25 (28)	7/10 (70)	3/5 (60)
Other autoimmune disorders^d^	9	5	3	2
CSF oligoclonal bands, (%)	18/24 (75)	4/15 (27)	4/9 (44)	7/7 (100)
Months from onset to GAD diagnosis, median (IQR)	4 (1–11)	36 (16–72)	19 (1–72)	12 (7–15)
Immunotherapy				
Steroids (+ IVIg)	4 (3)	2 (2)	0	5 (2)
Other combinations^e^	3	4	0	0

IQR: interquartile range; IVIg: intravenous immunoglobulin; T1DM: type 1 diabetes mellitus.

^a^ Coexistent stiff-person syndrome

^b^ Epilepsy (5 of them drug-resistant temporal lobe epilepsy)

^c^ Cerebellar ataxia

^d^ Pernicious anemia: 10 patients, celiac disease: 2, vitiligo: 5, psoriasis: 2, myasthenia: 2

^e^ IVIg or plasma exchange: 4; azathioprine: 2; IVIg + rituximab: 1.

### Standard Protocol Approvals and Patient Consents

All subjects gave written informed consent for the storage and use of serum and CSF samples for research purposes. Serum and CSF samples used in the study are deposited in the collection of biological samples named "Neuroinmunología" registered in the biobank of Institut d' Investigació Biomèdica August Pi i Sunyer (IDIBAPS), Barcelona, Spain. Animal handling procedures were approved by the Local Ethics Committee (99/1 University of Barcelona) and the Generalitat de Catalunya (1094/99), in accordance with the Directive 86/609/ EU of the European Commission. The study was approved by the Comité Ètic d’Investigació Clínica (CEIC) of Hospital Clinic.

### Immunohistochemistry of rat cerebellum

GAD-ab immunoreactivity was analyzed (serum screening dilution 1:500; CSF undiluted) using an avidin-biotin technique on paraformaldehyde-fixed frozen rat cerebellar sections as described previously [15]. To study the distribution of IgG subclasses of the antibody, the same immunohistochemistry technique was used after changing the secondary antibody by biotinylated mouse monoclonal antibodies to human IgG 1–4 subclasses (Sigma, St. Louis, MO) (dilutions: anti-IgG1 1:100, anti IgG2 1:200, anti-IgG3 1:200, and anti-IgG4 1:200) as described [16].

To study the presence of intrathecal synthesis of GAD-ab, the GAD-ab titers obtained by immunohistochemistry in paired samples of CSF and serum and the CSF/serum albumin index were used to calculate the index for intrathecal synthesis of GAD-ab as previously reported [1]. Values higher than 1, are a strong indicator of intrathecal synthesis of antibody-specific IgG [17].

### GAD65 ELISA

Levels of GAD65-ab were detected by ELISA (RSR Limited, UK) using a commercial kit following manufacturer’s specifications. Since GAD65 titres in neurologic syndromes are high, serums and CSF were titrated to determine the optimal dilution factor. Briefly, ELISA wells were seeded for 1 h with patients’ sera diluted 1:10000 or CSF diluted 1:200, followed by 1h incubation with GAD65 biotinylated protein, and 20 min incubation with streptavidin peroxidase. Colorimetric reaction was developed with 3,3’,5,5’-Tetramethylbenzidine for 20min, stopped with sulfuric acid and read at 450nm in a multiplate reader.

### Immunoblot

Human GAD65 (Diamyd, Stockholm, Sweden), GAD67 (Abnova, Taipei, Taiwan), and GABARAP (Abnova) recombinant proteins were electrophoretically separated and transferred to a polyvinylidene difluoride membrane and strips incubated with the patient's serum (1:1000 dilution), GAD65 (GAD-6, Hybridoma Bank, Iowa City, IA), GAD67 (Abcam, Cambridge, UK), or GABARAP (Abcam) commercial antibodies followed by biotinylated goat antihuman IgG or horse antimouse IgG (Vector Laboratories, Burlingame, CA) and developed with diaminobenzidine tetrahyrochloride [15]. In some experiments we used an enhanced chimioluminiscence technique (ECL Western Blotting System) following manufacturer’s instructions. The specificity of GAD65 and GAD67 commercial antibodies was confirmed with immunoblots with recombinant proteins and also with HEK293 cells (see below) expressing the corresponding antigens.

### Immunocytochemistry on HEK293 cells

To test the presence of antibodies against different synaptic antigens, HEK293 cells were transfected with plasmids containing human GAD65 or GAD67 (OriGene, Rockville, MD), the α1 and β3 subunits (co-transfected 1:1) of the GABAaR, GABARAP, α1 subunit of the GlyR, or gephyrin (co-transfected 1:1 with collybistin) as described [8,18–20]. All DNA sequences were purchased from OriGene except from those of GlyR, gephyrin and collybistin (a gift of Dr. RJ Harvey). Twenty-four hours after transfection, immunofluorescence on live (for the GABAaR and GlyR assays) or fixed (for the other assays) HEK293 cells was performed as described [5]. Briefly, HEK293 cells were incubated with patients’ serum (1:40) or CSF (1:5) combined with the corresponding primary commercial antibodies, GAD65 (GAD-6, Hybridoma Bank), GAD67 (Abcam), anti-GABAa receptor α1 subunit (Millipore Termecula, CA), anti-glycine receptor α1 subunit (Synaptic systems, Göttingen, Germany), or GABARAP (Abcam), followed by incubation with goat anti-human IgG Alexa Fluor 488 or goat anti-mouse IgG Alexa Fluor 594 (Life Technologies, Eugene, OR). When live HEK293 cells were used, they were fixed with 4% paraformaldehyde, and permeabilized with 0,3% triton X-100, after the incubation with patients’ and commercial primary antibodies. Results were photographed under fluorescence microscope (Zeiss Axioimager M2) using Zeiss Axiovision software (Zeiss, Oberkochen, Germany).

To validate the CBA for GAD65, 50 sera from patients with different neurological disorders (20 neurodegenerative, 15 NMDAR-ab, 10 anti-Hu, 5 other neuroimmunological disorders) and 17 from T1DM were used. None of the 50 sera were positive by this CBA and the sensitivity for T1DM depended on the titer: only 10/17 T1DM sera with higher antibody titer (1822–7900 U/ml by RIA) showed reactivity against GAD65. This finding indicates that the CBA for GAD65, like rat immunohistochemistry, is not sensitive enough to detect low levels of GAD65-ab (within the range typical of T1DM) and that are identified by RIA.

### GAD65 epitope analysis

We designed plasmids to express the sequence of the N-terminal domain (Nt: aa 1–188), the central region containing the decarboxylase catalytic domain also termed pyridoxal-5’-phosphate (PLP) domain to indicate the cofactor used by GAD (aa 189–464), and the C-terminal domain (Ct: aa 465–585) from GAD65 (SC300136, OriGene). Customized sequences were ordered and commercially subcloned with NheI-NotI (GenScript, Hong Kong) into a pCMV6-AC-Myc-tagged plasmid (PS100003, OriGene) Resulting plasmids were transfected into HEK293 cells and immunocytochemistry studies were performed as described above. A commercial antibody against the Myc-Tag protein (Myc-Tag (9B11) mouse mAb, Cell Signaling Technology) was used to confirm the correct expression of each construct.

### Analysis of antibodies against neuronal surface antigens

Antibodies against neuronal surface antigens were detected by immunohistochemistry of rat brain and immunofluorescence of rat hippocampal and cerebellar neuronal cultures as previously reported [5,21,22].

### Study of internalization of GAD IgG

To determine if GAD-ab are internalized in vivo, GAD-ab-positive CSF samples diluted 1:25 were incubated with live rat hippocampal neurons for 24h at 37°C. Cells were then washed with fresh neurobasal media and incubated with unlabeled anti-human IgG antibody (Jackson Immunoresearch, West Grove, PA) at 1:5 dilution for 1h at 37°C (to block detection of any specific or non-specific human IgG binding to the neuronal cell surface). After extensive washing, neurons were fixed for 5min with 4% paraformaldehyde, permeabilized with 0.1% Triton X-100 and incubated for 1h with Alexa Fluor 488 anti-human IgG. As positive control of the IgG internalization we used a NMDAR-ab-positive CSF. Results were photographed under confocal microscope (Zeiss LSM710) and analyzed with Zen software (Zen 2012 black edition 8.0, Zeiss).

### Statistical analysis

Non-parametric tests (Fisher exact, W Wilcoxon) were used when the application conditions for parametric tests (χ^2^, ANOVA) were violated. As ELISA values did not follow a normal distribution, log-transformation was used to normalize them prior to statistical analysis. Statistical software (Stata version 13; StataCorp) was used for the analyses and a *p* value < 0.05 was considered significant.

## Results

**Fig 1:**
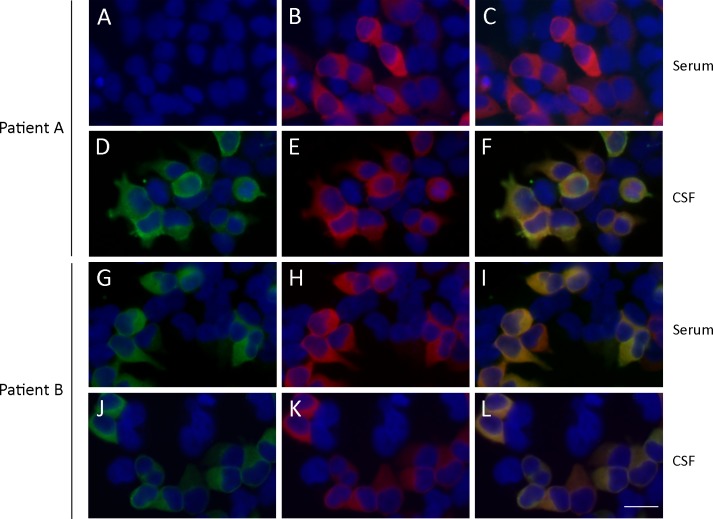
Reactivity of serum and CSF samples with HEK293 cells expressing GAD67. Fixed HEK293 cells transfected to express GAD67 were incubated with serum and CSF of patient A and B (in green) and a commercial antibody against GAD67 (in red). The nuclei of the cells are stained with 4′,6-diamidino-2-phenylindole (DAPI). The merged reactivity is shown in panels C, F, I, and L. The CSF, but not the serum, of patient A immunoreacted against GAD67, whereas the serum and CSF of patient B were both positive. Scale bar = 20μm.

By CBA, serum from the 106 (100%) patients had GAD65-ab and 88% GAD67-ab. No differences were observed in the frequency of GAD67-ab among the cerebellar ataxia, SPS, LE, or epilepsy groups. By contrast all 61 CSF tested (100%) contained both GAD65- and GAD67-ab (Fig. 1). There were nine paired serum-CSF samples in which the serum was negative and the CSF positive for GAD67-ab. These samples were equally distributed among the four clinical groups. Immunohistochemical analysis of IgG subclasses showed that in all 106 patients the predominant GAD immunoreactivity was IgG1.

**Fig 2:**
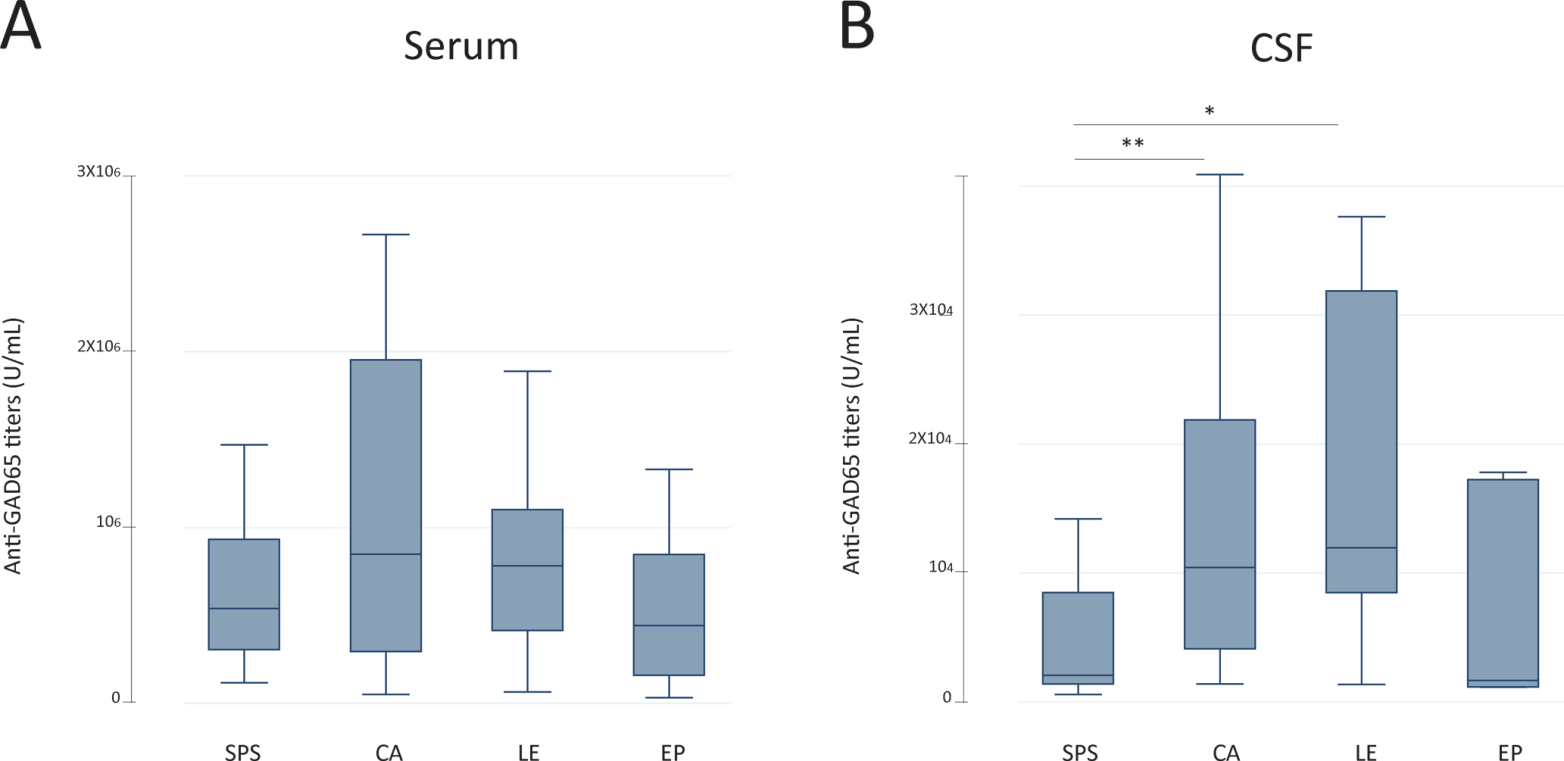
GAD65 antibody levels measured by ELISA in serum (A) and CSF (B) samples classified by neurological syndrome. CSF, but not serum, GAD65 antibody titres were significantly higher in the groups of ataxia and limbic encephalitis compared to those of stiff-person syndrome (median: 2.1 x10^3^ U/ml (interquartile range (IQR):1.4–8.5) in SPS vs. 10.4 x10^3^ U/ml (4.1–21.9) in CA; **p = 0.01 and 12 x10^3^ U/ml (8.5–31.9) in LE; *p = 0.02). SPS: stiff person syndrome; CA: cerebellar ataxia; LE: limbic encephalitis; EP: epilepsy.

**Fig 3:**
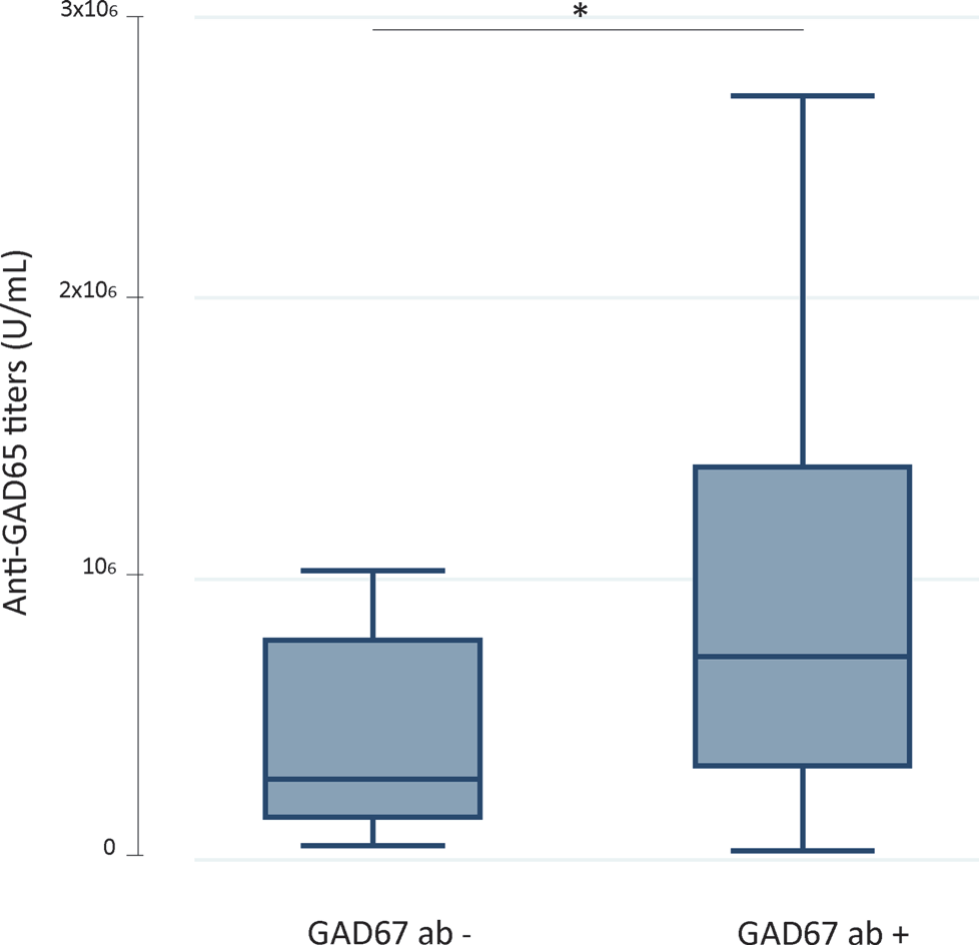
GAD65 antibody titres measured by ELISA in serum samples from patients with (n = 93) or without (n = 13) additional GAD67 antibodies. GAD65 antibody levels were higher in the group of patients with concurrent GAD67 antibodies. *p≤0.05.

Serum GAD65-ab levels tended to be higher in patients with cerebellar ataxia and LE (Fig. 2A). This trend was confirmed in the CSF where GAD65-ab levels were significantly higher in patients with cerebellar ataxia and LE compared to those of SPS patients (Fig. 2B). In addition, the median index of intrathecal synthesis of GAD65-ab in available paired serum/CSF was higher in 14 patients with cerebellar ataxia (median: 9.7 [IQR: 3.6–16.6]) compared to that of 11 with SPS (5.3 [2.3–17.4]); *p* = 0.38. The presence of serum GAD67-ab was associated with GAD65-ab titres (7.2 x10^5^ U/ml (3.3–14.0) vs. 2.9 x10^5^ U/ml (1.5–7.8) for patients with and without serum GAD67-ab respectively; *p* = 0.02; Fig. 3).

### Analysis of GAD-ab epitopes

**Fig 4:**
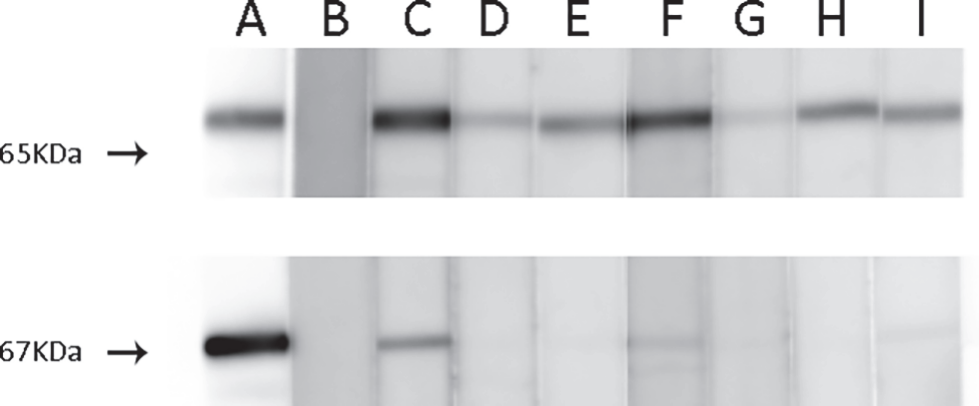
Immunoblot of purified GAD65 (upper panel) or GAD67 protein (lower panel). Strips were incubated with a commercial antibody (A), serum from a healthy individual (B), and serum from patients with cerebellar ataxia (C and D), stiff-person syndrome (E), limbic encephalitis (F and G) and epilepsy (H and I). All patients’ sera reacted against GAD65 but only a few recognized GAD67 despite that all immunoreacted with HEK293 cells transfected with GAD67; this finding suggests that the recognized epitope is conformational. Both gels were run at the same time and blots were developed in parallel. The images were cropped to include the visible bands.

To elucidate whether GAD65- and GAD67-ab identify linear epitopes, all 106 serum samples were screened by immunoblot of purified GAD65 and GAD67 recombinant protein. All but two sera (98%) reacted with GAD65 whereas only 39 (42%) of the 93 sera with GAD67-ab assessed by the CBA were positive by immunoblot of GAD67 (Fig. 4). No correlation was found between the serum reactivity with GAD67 immunoblot and any specific neurological syndromes.

**Fig 5:**
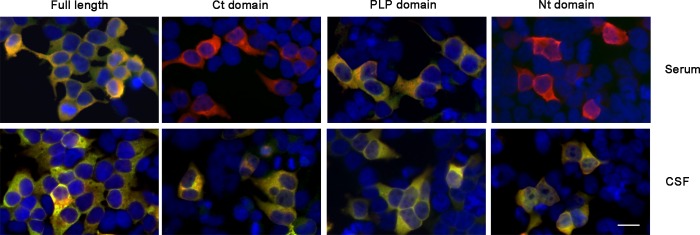
Study of GAD65 epitope immunoreactivity. Fixed HEK293 cells transfected to express full length GAD65, C-terminal (Ct), middle (PLP) and N-terminal (Nt) domains (all expressing an N-terminal myc tag) were incubated with patient serum (upper panels) or CSF (lower panels) (green) and a commercial antibody against myc tag (red). The nuclei of the cells are stained with 4′,6-diamidino-2-phenylindole (DAPI) (blue). The merged reactivity is shown in yellow. The serum sample reacts against full length and PLP domain, whereas the CSF sample shows a broader reactivity, staining the full length and three GAD65 domains. Scale bar = 20μm.

To investigate the antigenic region of GAD65 recognized by GAD65-ab of patients with the different neurological syndromes, we produced constructs of the three different domains of GAD65: Nt, PLP, and Ct. After demonstrating that the subcloning process did not induce a decrease in GAD65 immunoreactivity (data not shown), we then tested the reactivity of all sera and CSF samples on HEK293 cells expressing each of the three constructs. In 46% of patients serum GAD65-ab recognized only one domain, whereas 74% of CSF GAD65-ab recognized all three domains (*p* < 0.001) (Fig. 5). The most frequently recognized immunodominant region in serum was in the PLP domain; 93% of the sera showed reactivity against this region, including those with the lower titre of GAD65-ab. No correlation was noted between GAD65-ab levels and reactivity with any particular epitope. Serum of patients with LE were more likely to react with the Nt domain (69% vs. 29% for the rest of the patients; *p* = 0.002), whereas epileptic patients showed more reactivity against the Ct domain (67% vs. 38% for the other 3 groups; *p* = 0.04). We did not find these differences in CSF, since most of the samples recognized all three domains. No other epitope-region differences were seen among clinical phenotypes. The distribution of frequencies of the number of domains recognized by serum GAD65-ab was similar between the group of sera with or without GAD67-ab, indicating that the presence of GAD67-ab was not related to the extent of the reactivity against GAD65.

### Presence of additional antibodies against the GABAergic inhibitory synapsis

All 106 serum and 61 CSF samples were examined by immunofluorescence with live rat hippocampal neurons (sera from cerebellar ataxia and stiff-person syndrome cases where also tested in cultures of cerebellar neurons), HEK293 cells expressing α1/β3 subunits of GABAaR, α1 subunit of GlyR, gephyrin or GABARAP. We found six patients with GABAaR antibodies (1 cerebellar ataxia, 2 SPS and 3 epilepsy, all in serum, CSF not available) and six with GlyR antibodies (4 cerebellar and 2 epilepsy, all in serum, negative in the four CSF available). All samples had low titres (<1:200) of the antibodies. No additional antibodies against neuronal surface antigens were detected in neuronal cultures. We did not find any patient with GABARAP or gephyrin-ab. This result was confirmed by immunoblot using GABARAP recombinant proteins that were tested with serum from 32 patients with SPS (not shown).

### Internalization of GAD-ab

**Fig 6:**
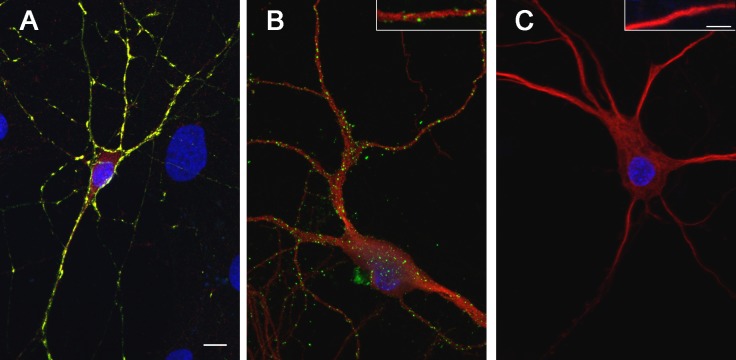
Study of anti-GAD IgG internalization in cultures of dissociated rat hippocampal neurons that express GAD65. Panel A demonstrates that neurons express GAD65; in this experiment neurons were permeabilized and incubated with a patient’s CSF with GAD65-ab (green) and a commercial GAD65-ab (red); the co-localization of reactivity with GAD65 is shown in yellow. Panels B and C show the experiment of internalization; in B, live neurons were incubated with CSF of a patient with anti-NMDAR antibodies, and in C with the CSF of a patient with GAD65 antibodies for 24h at room temperature. After blocking the extracellular IgG binding with a secondary antibody without fluorescent tag, the neurons were washed, fixed and permeabilized and the internalized IgG was determined with a secondary anti-human IgG antibody with a fluorescence tag (green), or with an antibody against MAP2 (red). Insets show dendrites at higher magnification. Only the CSF of the patient with IgG antibodies against NMDAR showed IgG internalization, as previously reported (Hughes *et al*. 2010), and used here as a control. Scale bar = 10μm, insert scale bar = 2,5μm.

If we postulate that GAD65 antibodies are pathogenic, they should reach the antigen, which is intracellular, and for this the antibodies need to be internalized. For this experiment we first confirmed that the cultured neurons used contained GAD65. Therefore, cultured neurons were first fixed, permeabilized and incubated with a commercial GAD-6 monoclonal antibody along with patients’ CSF. This preliminary study indicated that the neurons contained intracytoplasmic GAD65 and that both, the commercial antibodies and patients’ CSF antibodies were able to react with the antigen when the cell membrane was permeabilized (Fig. 6, panel A). Subsequently, we examined if patients’ antibodies were able to react with live neurons (membrane not permeabilized). For this experiment live rat hippocampal neurons were incubated for 24 hours with GAD65 antibodies contained in the CSF of four patients with cerebellar ataxia, four with SPS, three with LE and five with epilepsy; the same experiment using CSF with NMDAR antibodies from a patient with anti-NMDA receptor encephalitis served as control of IgG internalization. No intracellular staining was detected in any of the four groups of patients with GAD-ab whereas IgG internalization was observed with the CSF of the patient with NMDAR-ab as previously reported [23] (Fig. 6). Overall, these set of studies demonstrate that patients ´ GAD65 antibodies only react with GAD65 when the membrane is permeabilized but not in live neurons.

## Discussion

The current study provides for first time a comprehensive overview of the humoral immune responses present in serum and CSF of patients with neurological disorders associated with GAD-ab. Our data show several important immunological features; 1) the humoral immune response against GAD is different in CSF and serum. In the CSF the antibodies target more domains of GAD65 and also immunoreact with GAD67, 2) GAD65-ab titres were higher in the CSF of patients with LE or cerebellar ataxia compared to those with SPS, 3) the frequency of additional antibodies against antigens of the GABAergic inhibitory synapses is low across all neurological syndromes, and 4) GAD-ab are not internalized by primary neuronal cultures suggesting that GAD-ab are not directly pathogenic.

A novel finding of our study is that the humoral response to GAD was different in CSF and serum. All patients regardless of the associated neurological syndrome had GAD67-ab in the CSF, including 9/61 (15%) patients whose serum was negative for these antibodies. In addition, CSF GAD65-ab usually recognized epitopes present in the three domains examined. These data along with the frequent detection of CSF oligoclonal bands [24,25] and specific intrathecal synthesis of GAD65-ab [1,17,26] strongly suggest that the presence of CSF GAD65-ab is not only a result of passive antibody transfer from serum to CSF, but they are part of an active immune response orchestrated in the CNS compartment.

Although we did not identify differences in the GAD65 epitope repertoire among the four neurological syndromes examined, CSF levels of GAD65-ab were lower in patients with SPS than in those with cerebellar ataxia or LE which unlike SPS, commonly associate with neuronal loss[6,27]. In a previous series of five patients with cerebellar ataxia, the CSF GAD65-ab titres were similar to those of 10 patients with SPS. However, patients with cerebellar ataxia showed a 2.5-fold higher intrathecal production of GAD65-ab compared to SPS[28] as we have also found in the current study.

Analysis of GAD65-ab epitopes has been previously done in patients with T1DM and SPS but not in other neurological syndromes. In T1DM, GAD65-ab mostly recognize conformational epitopes in the PLP and Ct domains [29,30]. Initial epitope studies in cohorts of SPS found that the strongest reactivity with GAD65 depended on two discontinuous domains in the Ct, and in addition most sera recognized at least one epitope in the Nt domain [31]. Other authors also described a dominant linear epitope located in the Nt domain [32,33]. In the present study, the PLP domain was the GAD65 region most frequently recognized by serum GAD65-ab with no differences among neurological syndromes. Our findings are similar to those of a recent study that using a luciferase immunoprecipitation technique and GAD65 sub-fragments deletion mutants showed that the central region containing the decarboxylase catalytic domain was highly immunoreactive with sera of all patients with SPS [34]. We did not find a significant difference in GAD65 immunodominant regions in patients with the four indicated neurological syndromes. Our analysis of immunodominant domains included three large GAD65 deletion constructs spanning the entire molecule. This analysis could have missed smaller protein segments containing epitopes specific for a particular neurological syndrome [35].

We did not find antibodies against gephyrin and GABARAP. Gephyrin is a protein involved in clustering both GlyR and GABAaR [36] and antibodies to this protein were described in a single patient with SPS and mediastinal cancer [37]. Antibodies to GABARAP, a protein that stabilizes GABAaR in the membrane [38], were described in 70% of a cohort of 27 patients with SPS and GAD65-ab [39]. It is unclear why we did not detect GABARAP-ab but the study published in 2006 has never been replicated. The methods of detection were different; while GABARAP-ab were initially detected by immunoprecipitation and immunoblot, we have used CBA for all our patients and immunoblot with recombinant GABARAP in a subgroup randomly selected. We cannot rule out that our techniques were less sensitive but if this was the case the levels of GABARAP antibodies should be very low.

The recent discovery of antibodies against cell-surface or synaptic receptors in patients with autoimmune encephalitis, along with data supporting their pathogenicity and sometimes coexistence with GAD-ab, led us to investigate whether additional cell-surface antibodies could offer an explanation for the syndrome diversity. In the current study we used the same approach that resulted in the discovery of 9 of 11 cell surface or synaptic receptor autoantigens [40] and only identified 12 cases (11%) with these antibodies, all directed against known target antigens (six GABAaR and six GlyR) and with a similar distribution among clinical phenotypes. These findings are therefore different from those of a recent study [41] that identified antibodies against unknown cell surface antigens in patients with GAD65-ab. We did not find coexisting GlyR-ab in patients with SPS; this is in contrast with a previous report that showed that 15% of patients had coexisting GAD65 and GlyR antibodies [42]. If different serum dilutions or other technical details are responsible for this difference is unclear; we used a similar live cell-based assay but the serum dilution was not provided in the previous study[42].

Lastly, the pathogenic role of GAD-ab has been subjected to debate [3]. In vitro studies have demonstrated that GAD-ab from SPS patients, but not from diabetic patients, inhibit the enzymatic activity of GAD65 [43]. Intrathecal and intracerebellar injections of IgG purified from GAD65-ab-positive samples of patients with SPS or cerebellar ataxia, or human monoclonal antibodies to GAD65, induced clinical symptoms and neurophysiological changes similar to those seen in patients [10,11,35,44]. However, these experiments failed to unambiguously demonstrate that the observed changes were directly related to the interaction of the antibodies with GAD65. Similarly, active immunization experiments with GAD65 did not cause neurological dysfunction in immunized animals [45,46]. GAD65 is an intracellular antigen and therefore an essential experiment is the demonstration that GAD-ab are internalized and reach the antigen. Previous studies showed that human monoclonal antibodies to GAD65 were internalized by cells from an immortalized rat CNS cell line [44] or into neurons surrounding the area of injection into rat cerebral cortex[47]. However, none of these studies showed that the antibodies reacted with intracellular GAD in vivo [44,47]. Our in vitro experiments failed to show that GAD-ab are internalized in cultures of neurons that express GAD65 casting doubt on the antibody pathogenicity. These experiments are nor definite proof that GAD-ab are not pathogenic; however, to resolve this issue future studies should unambiguously demonstrate that GAD-ab can reach the target antigen, visualizing the binding with high resolution confocal microscopy, and determining if the potential effects on GAD correlate with symptoms using appropriate animal models, as recently demonstrated for NMDAR antibodies in patients with anti-NMDAR encephalitis [48].

## Conclusions

The present study emphasizes the importance of studying the CSF of patients with CNS syndromes suspected to be mediated by GAD autoimmunity because the repertoire of antibodies to different immunodominant regions is wider in the CNS than systemically. We did not find a specific immunodominant region or associated antibody that could be used as a biomarker of a particular neurological anti-GAD-associated syndrome. Moreover, GAD-ab are not internalized in live neurons, raising doubts about the direct pathogenicity of the antibodies.

## References

[1] Saiz A, Blanco Y, Sabater L, González F, Bataller L, Casamitjana R, et al Spectrum of neurological syndromes associated with glutamic acid decarboxylase antibodies: diagnostic clues for this association. Brain 2008;131:2553–63. 10.1093/brain/awn183 18687732

[2] Ali F, Rowley M, Jayakrishnan B, Teuber S, Gershwin ME, Mackay IR. Stiff-person syndrome (SPS) and anti-GAD-related CNS degenerations: protean additions to the autoimmune central neuropathies. Journal of Autoimmunity 2011;37:79–87. 10.1016/j.jaut.2011.05.005 21680149

[3] Alexopoulos H, Dalakas MC. Immunology of stiff person syndrome and other GAD-associated neurological disorders. Expert Review of Clinical Immunology 2013;9:1043–53. 10.1586/1744666X.2013.845527 24168411

[4] Solimena M, Folli F, Denis-Donini S, Comi GC, Pozza G, De Camilli P, et al Autoantibodies to glutamic acid decarboxylase in a patient with stiff-man syndrome, epilepsy, and type I diabetes mellitus. The New England Journal of Medicine 1988;318:1012–20. 3281011 10.1056/NEJM198804213181602

[5] Lancaster E, Lai M, Peng X, Hughes E, Constantinescu R, Raizer J, et al Antibodies to the GABA(B) receptor in limbic encephalitis with seizures: case series and characterisation of the antigen. Lancet Neurology 2010;9:67–76. 10.1016/S1474-4422(09)70324-2 19962348 PMC2824142

[6] Malter MP, Helmstaedter C, Urbach H, Vincent A, Bien CG. Antibodies to glutamic acid decarboxylase define a form of limbic encephalitis. Annals of Neurology 2010;67:470–8. 10.1002/ana.21917 20437582

[7] Dalakas MC, Fujii M, Li M, Lutfi B, Kyhos J, McElroy B. High-dose intravenous immune globulin for stiff-person syndrome. The New England Journal of Medicine 2001;345:1870–6. 11756577 10.1056/NEJMoa01167

[8] Ariño H, Gresa-Arribas N, Blanco Y, Martínez-Hernández E, Sabater L, Petit-Pedrol M, et al Cerebellar ataxia and glutamic acid decarboxylase antibodies: immunologic profile and long-term effect of immunotherapy. JAMA Neurology 2014;71:1009–16. 10.1001/jamaneurol.2014.1011 24934144 PMC4841264

[9] Rakocevic G, Raju R, Dalakas MC. Anti-glutamic acid decarboxylase antibodies in the serum and cerebrospinal fluid of patients with stiff-person syndrome: correlation with clinical severity. Archives of Neurology 2004;61:902–4. 15210528 10.1001/archneur.61.6.902

[10] Manto M-U, Laute M-A, Aguera M, Rogemond V, Pandolfo M, Honnorat J. Effects of anti-glutamic acid decarboxylase antibodies associated with neurological diseases. Annals of Neurology 2007;61:544–51. 17600364 10.1002/ana.21123

[11] Hansen N, Grünewald B, Weishaupt A, Colaço MN, Toyka K V, Sommer C, et al Human Stiff person syndrome IgG-containing high-titer anti-GAD65 autoantibodies induce motor dysfunction in rats. Experimental Neurology 2013;239:202–9. 10.1016/j.expneurol.2012.10.013 23099416

[12] Graus F, Delattre JY, Antoine JC, Dalmau J, Giometto B, Grisold W, et al Recommended diagnostic criteria for paraneoplastic neurological syndromes. Journal of Neurology, Neurosurgery, and Psychiatry 2004;75:1135–40. 15258215 10.1136/jnnp.2003.034447 PMC1739186

[13] McKeon A, Robinson MT, McEvoy KM, Matsumoto JY, Lennon VA, Ahlskog JE, et al Stiff-man syndrome and variants: clinical course, treatments, and outcomes. Archives of Neurology 2012;69:230–8. 10.1001/archneurol.2011.991 22332190

[14] Gultekin SH, Rosenfeld MR, Voltz R, Eichen J, Posner JB, Dalmau J. Paraneoplastic limbic encephalitis: neurological symptoms, immunological findings and tumour association in 50 patients. Brain: A Journal of Neurology 2000;123 (Pt 7):1481–94. 10869059 10.1093/brain/123.7.1481

[15] Saiz A, Arpa J, Sagasta A, Casamitjana R, Zarranz JJ, Tolosa E, et al Autoantibodies to glutamic acid decarboxylase in three patients with cerebellar ataxia, late-onset insulin-dependent diabetes mellitus, and polyendocrine autoimmunity. Neurology 1997;49:1026–30. 9339684 10.1212/wnl.49.4.1026

[16] Bernal F, Shams’ili S, Rojas I, Sanchez-Valle R, Saiz A, Dalmau J, et al Anti-Tr antibodies as markers of paraneoplastic cerebellar degeneration and Hodgkin’s disease. Neurology 2003;60:230–4. 12552036 10.1212/01.wnl.0000041495.87539.98

[17] Dalakas MC, Li M, Fujii M, Jacobowitz DM. Stiff person syndrome: quantification, specificity, and intrathecal synthesis of GAD65 antibodies. Neurology 2001;57:780–4. 11552003 10.1212/wnl.57.5.780

[18] Boronat A, Sabater L, Saiz A, Dalmau J, Graus F. GABA(B) receptor antibodies in limbic encephalitis and anti-GAD-associated neurologic disorders. Neurology 2011;76:795–800. 10.1212/WNL.0b013e31820e7b8d 21357831 PMC3053332

[19] McKeon A, Martinez-Hernandez E, Lancaster E, Matsumoto JY, Harvey RJ, McEvoy KM, et al Glycine receptor autoimmune spectrum with stiff-man syndrome phenotype. JAMA Neurology 2013;70:44–50. 10.1001/jamaneurol.2013.574 23090334 PMC3718477

[20] Petit-Pedrol M, Armangue T, Peng X, Bataller L, Cellucci T, Davis R, et al Encephalitis with refractory seizures, status epilepticus, and antibodies to the GABAA receptor: a case series, characterisation of the antigen, and analysis of the effects of antibodies. Lancet Neurology 2014;13:276–86. 10.1016/S1474-4422(13)70299-0 24462240 PMC4838043

[21] Ances BM, Vitaliani R, Taylor RA, Liebeskind DS, Voloschin A, Houghton DJ, et al Treatment-responsive limbic encephalitis identified by neuropil antibodies: MRI and PET correlates. Brain 2005;128:1764–77. 15888538 10.1093/brain/awh526 PMC1939694

[22] Sabater L, Höftberger R, Boronat A, Saiz A, Dalmau J, Graus F. Antibody repertoire in paraneoplastic cerebellar degeneration and small cell lung cancer. PloS One 2013;8:e60438 10.1371/journal.pone.0060438 23536908 PMC3607586

[23] Hughes EG, Peng X, Gleichman AJ, Lai M, Zhou L, Tsou R, et al Cellular and synaptic mechanisms of anti-NMDA receptor encephalitis. The Journal of Neuroscience 2010;30:5866–75. 10.1523/JNEUROSCI.0167-10.2010 20427647 PMC2868315

[24] Honnorat J, Saiz A, Giometto B, Vincent A, Brieva L, de Andres C, et al Cerebellar ataxia with anti-glutamic acid decarboxylase antibodies: study of 14 patients. Archives of Neurology 2001;58:225–30. 11176960 10.1001/archneur.58.2.225

[25] Skorstad G, Hestvik AL, Torjesen P, Alvik K, Vartdal F, Vandvik B, et al GAD65 IgG autoantibodies in stiff person syndrome: clonality, avidity and persistence. European Journal of Neurology 2008;15:973–80. 10.1111/j.1468-1331.2008.02221.x 18637036

[26] Jarius S, Stich O, Speck J, Rasiah C, Wildemann B, Meinck HM, et al Qualitative and quantitative evidence of anti-glutamic acid decarboxylase-specific intrathecal antibody synthesis in patients with stiff person syndrome. Journal of Neuroimmunology 2010;229:219–24. 10.1016/j.jneuroim.2010.07.019 20813415

[27] Ishida K, Mitoma H, Wada Y, Oka T, Shibahara J, Saito Y, et al Selective loss of Purkinje cells in a patient with anti-glutamic acid decarboxylase antibody-associated cerebellar ataxia. Journal of Neurology, Neurosurgery, and Psychiatry 2007;78:190–2. 17119008 10.1136/jnnp.2006.091116 PMC2077656

[28] Rakocevic G, Raju R, Semino-Mora C, Dalakas MC. Stiff person syndrome with cerebellar disease and high-titer anti-GAD antibodies. Neurology 2006;67:1068–70. 17000981 10.1212/01.wnl.0000237558.83349.d0

[29] Richter W, Shi Y, Baekkeskov S. Autoreactive epitopes defined by diabetes-associated human monoclonal antibodies are localized in the middle and C-terminal domains of the smaller form of glutamate decarboxylase. Proceedings of the National Academy of Sciences of the United States of America 1993;90:2832–6. 7681990 10.1073/pnas.90.7.2832 PMC46190

[30] Fenalti G, Hampe CS, Arafat Y, Law RHP, Banga JP, Mackay IR, et al COOH-terminal clustering of autoantibody and T-cell determinants on the structure of GAD65 provide insights into the molecular basis of autoreactivity. Diabetes 2008;57:1293–301. 10.2337/db07-1461 18184926

[31] Butler MH, Solimena M, Dirkx R, Hayday A, De Camilli P. Identification of a dominant epitope of glutamic acid decarboxylase (GAD-65) recognized by autoantibodies in stiff-man syndrome. The Journal of Experimental Medicine 1993;178:2097–106. 8245784 10.1084/jem.178.6.2097 PMC2191306

[32] Kim J, Namchuk M, Bugawan T, Fu Q, Jaffe M. Higher autoantibody levels and recognition of a linear NH2-terminal epitope in the autoantigen GAD65, distinguish stiff-man syndrome from insulin-dependent diabetes mellitus. The Journal of Experimental Medicine 1994;180:595–606. 7519242 10.1084/jem.180.2.595 PMC2191592

[33] Raju R, Foote J, Banga JP, Hall TR, Padoa CJ, Dalakas MC, et al Analysis of GAD65 autoantibodies in Stiff-Person syndrome patients. Journal of Immunology 2005;175:7755–62. 16301686 10.4049/jimmunol.175.11.7755

[34] Burbelo PD, Groot S, Dalakas MC, Iadarola MJ. High definition profiling of autoantibodies to glutamic acid decarboxylases GAD65/GAD67 in stiff-person syndrome. Biochemical and Biophysical Research Communications 2008;366:1–7. 18047830 10.1016/j.bbrc.2007.11.077 PMC2215321

[35] Manto MU, Hampe CS, Rogemond V, Honnorat J. Respective implications of glutamate decarboxylase antibodies in stiff person syndrome and cerebellar ataxia. Orphanet Journal of Rare Diseases 2011;6:3 10.1186/1750-1172-6-3 21294897 PMC3042903

[36] Tyagarajan SK, Fritschy J-M. Gephyrin: a master regulator of neuronal function? Nature Reviews Neuroscience 2014;15:141–56. 10.1038/nrn3670 24552784

[37] Butler MH, Hayashi A, Ohkoshi N, Villmann C, Becker CM, Feng G, et al Autoimmunity to gephyrin in Stiff-Man syndrome. Neuron 2000;26:307–12. 10839351 10.1016/s0896-6273(00)81165-4

[38] Chen Z-W, Olsen RW. GABAA receptor associated proteins: a key factor regulating GABAA receptor function. Journal of Neurochemistry 2007;100:279–94. 17083446 10.1111/j.1471-4159.2006.04206.x

[39] Raju R, Rakocevic G, Chen Z, Hoehn G, Semino-Mora C, Shi W, et al Autoimmunity to GABAA-receptor-associated protein in stiff-person syndrome. Brain 2006;129:3270–6. 16984900 10.1093/brain/awl245

[40] Leypoldt F, Armangue T, Dalmau J. Autoimmune encephalopathies. Annals of the New York Academy of Sciences 2014 [Epub ahead of print]. 10.1111/nyas.12553 PMC4363225 25315420

[41] Chang T, Alexopoulos H, McMenamin M, Carvajal-González A, Alexander SK, Deacon R, et al Neuronal Surface and Glutamic Acid Decarboxylase Autoantibodies in Nonparaneoplastic Stiff Person Syndrome. JAMA Neurology 2013;70:1140–9. 23877118 10.1001/jamaneurol.2013.3499 PMC6055982

[42] Alexopoulos H, Akrivou S, Dalakas MC. Glycine receptor antibodies in stiff-person syndrome and other GAD-positive CNS disorders. Neurology 2013;81:1962–4. 10.1212/01.wnl.0000436617.40779.65 24174585

[43] Dinkel K, Meinck HM, Jury KM, Karges W, Richter W. Inhibition of gamma-aminobutyric acid synthesis by glutamic acid decarboxylase autoantibodies in stiff-man syndrome. Annals of Neurology 1998;44:194–201. 9708541 10.1002/ana.410440209

[44] Hampe CS, Petrosini L, De Bartolo P, Caporali P, Cutuli D, Laricchiuta D, et al Monoclonal antibodies to 65kDa glutamate decarboxylase induce epitope specific effects on motor and cognitive functions in rats. Orphanet Journal of Rare Diseases 2013;8:82 10.1186/1750-1172-8-82 23738610 PMC3680042

[45] Han G, Li Y, Wang J, Wang R, Chen G, Song L, et al Active tolerance induction and prevention of autoimmune diabetes by immunogene therapy using recombinant adenoassociated virus expressing glutamic acid decarboxylase 65 peptide GAD(500–585). Journal of Immunology 2005;174:4516–24. 15814672 10.4049/jimmunol.174.8.4516

[46] Chang T, Alexopoulos H, Pettingill P, McMenamin M, Deacon R, Erdelyi F, et al Immunization against GAD Induces Antibody Binding to GAD-Independent Antigens and Brainstem GABAergic Neuronal Loss. PloS One 2013;8:e72921 10.1371/journal.pone.0072921 24058450 PMC3776810

[47] Vega-Flores G, Rubio SE, Jurado-Parras MT, Gómez-Climent MÁ, Hampe CS, Manto M, et al The GABAergic septohippocampal pathway is directly involved in internal processes related to operant reward learning. Cerebral Cortex 2014;24:2093–107. 10.1093/cercor/bht060 23479403 PMC4441070

[48] Planagumà J, Leypoldt F, Mannara F, Gutiérrez-Cuesta J, Martín-García E, Aguilar E, et al Human N-methyl D-aspartate receptor antibodies alter memory and behaviour in mice. Brain 2015;138:94–109. 10.1093/brain/awu310 25392198 PMC4285189

